# Climate Justice Implications of Natech Disasters: Excess Contaminant Releases during Hurricanes on the Texas Gulf Coast

**DOI:** 10.1021/acs.est.3c10797

**Published:** 2024-07-30

**Authors:** Alique G. Berberian, Rachel Morello-Frosch, Seigi Karasaki, Lara J. Cushing

**Affiliations:** † Department of Environmental Health Sciences, University of California, Los Angeles, California 90095, United States; ‡ Department of Environmental Science, Policy and Management and School of Public Health, University of California, Berkeley, California 94720, United States; § Energy and Resources Group, University of California, Berkeley, California 94720, United States

**Keywords:** climate change, natech, climate resilience, tropical cyclone, environmental justice

## Abstract

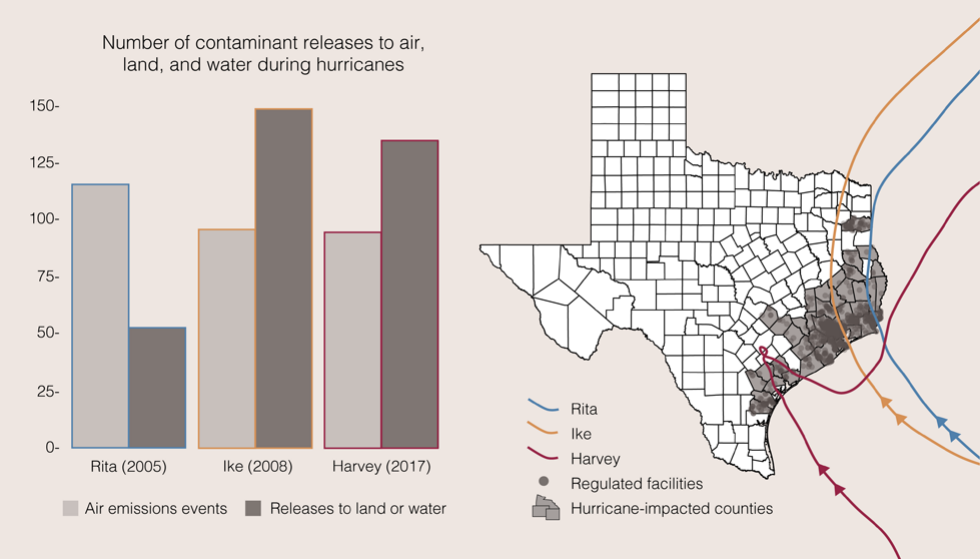

Extreme weather events are becoming more severe due to climate change, increasing the risk of contaminant releases from hazardous sites disproportionately located in low-income communities of color. We evaluated contaminant releases during Hurricanes Rita, Ike, and Harvey in Texas and used regression models to estimate associations between neighborhood racial/ethnic composition and residential proximity to hurricane-related contaminant releases. Two-to-three times as many excess releases were reported during hurricanes compared to business-as-usual periods. Petrochemical manufacturing and refineries were responsible for most air emissions events. Multivariable models revealed sociodemographic disparities in likelihood of releases; compared to neighborhoods near regulated facilities without a release, a one-percent increase in Hispanic residents was associated with a 5 and 10% increase in the likelihood of an air emissions event downwind and within 2 km during Hurricanes Rita and Ike (odds ratio and 95% credible interval= 1.05 [1.00, 1.13], combined model) and Harvey (1.10 [1.00, 1.23]), respectively. Higher percentages of renters (1.07 [1.03, 1.11], combined Rita and Ike model) and rates of poverty (1.06 [1.01, 1.12], Harvey model) were associated with a higher likelihood of a release to land or water, while the percentage of Black residents (0.94 [0.89, 1.00], Harvey model) was associated with a slightly lower likelihood. Population density was consistently associated with a decreased likelihood of a contaminant release to air, land, or water. Our findings highlight social inequalities in the risks posed by natural–technological disasters that disproportionately impact Hispanic, renter, low-income, and rural populations.

## Abstract 2

Limited research exists on the environmental justice implications of natechs due to extreme weather. This study evaluates the magnitude of contaminant releases during hurricanes affecting the Texas Gulf Coast and the associated risks to marginalized populations.

## Introduction

The severity and frequency of tropical cyclones are increasing in parts of the United States (US) due to climate change.^1−3^ The frequency of jointly occurring precipitation and storm surge during storm events is also increasing in many US cities, exacerbating overall flood risk.^4−6^ Low-income communities and people of color are disproportionately impacted by hurricanes and floods, leading to concerns that climate change will further exacerbate existing environmental health disparities.^7^ National flood risk assessments have found that socially vulnerable and economically disadvantaged populations are more likely to live in flood zones.^8,9^ After Hurricane Katrina (2005), many studies documented disproportionate flooding, displacement, and adverse health outcomes among low-income and Black residents of New Orleans.^10−13^ Research following Hurricane Sandy (2010) identified socioeconomic disparities in flood exposure in New York City and Long Island.^14^ Similarly, neighborhoods with higher proportions of Black, Hispanic, and socioeconomically deprived residents in the Greater Houston area experienced a significantly greater extent of flooding compared to White and high-socioeconomic status (SES) residents during Hurricane Harvey (2017).^15,16^

Extreme weather also poses risks to industrial sites like chemical plants, refineries, hazardous waste treatment facilities, and legacy cleanup sites that manufacture, use, or store hazardous materials.^17,18^ Flooding, strong winds, tornadoes, and storm surges can damage infrastructure, cause power failures and equipment malfunctions, and prevent personnel access to industrial sites, which may lead to natural–technological (natech) disasters^19^—cascading events in which natural hazards trigger technological accidents that result in contaminant releases. Impacts from natech events have environmental and health equity implications. For example, oil spills from storage tanks can contaminate water sources, and releases of toxic air contaminants from chemical plants can cause acute changes to ambient air quality and increase the risk for adverse health effects. Because people of color and of low SES in the US are more likely to live near industrial sites,^20^ natech disasters are likely to disproportionately impact marginalized communities.

We assessed the environmental justice implications of excess contaminant releases to air, water, and land during major hurricanes affecting industrialized regions of the Texas Gulf Coast over the last two decades. The Texas Gulf Coast is prone to climate change-related extreme and frequent weather events,^21−23^ is rapidly urbanizing, and is a major hub for the petrochemical industry, making the region highly vulnerable to natech disasters. The Houston Ship Channel industrial corridor alone has 866 industrial facility parcels, 5 oil refineries, and more than 3400 aboveground storage tanks housing hazardous materials.^24^ A large proportion of Houston’s urban development exists in the current flood-prone zone,^25^ and the city’s lack of zoning regulations has resulted in many communities living in close proximity to hazardous sites,^26^ raising concerns about toxic exposures to chemicals among fenceline communities during flood events.^27^ Several environmental justice studies in Houston have documented socioeconomic and racial/ethnic disparities in risks to environmental hazards and toxic exposures, including from air pollution, hazardous waste facilities, and flooding, suggesting existing vulnerabilities in Houston communities that are likely to be exacerbated during hurricanes and natech events.^27−30^

We include Hurricanes Rita (2005), Ike (2008), and Harvey (2017) because these hurricanes all made landfall along the Texas Gulf Coast, were designated as Category 4–5 at their peak (determined by their sustained wind speed and destructive power), and passed through or near Houston. Hurricane Rita made landfall near the Texas-Louisiana border in September 2005, less than 4 weeks after Hurricane Katrina devastated southeast Louisiana and coastal Mississippi. Impacts from these consecutive hurricanes prompted the shutdown of almost all offshore crude oil and natural gas production for several days, as well as destroyed and caused extensive damage to offshore oil and gas infrastructure, including pipelines, platforms, and rigs.^31,32^ It was estimated that more than 3000 of the 4000 platforms and 22 000 of the 33 000 miles of pipelines in the Gulf were in the direct path of either Hurricane Katrina or Rita.^33^ Hurricane Ike made landfall on Galveston Island in September 2008, 2 weeks after Hurricane Gustav struck southeast Louisiana, and generated significant storm surge (15–20 feet in parts of Chambers County), despite being ranked a moderately intense (Category 2) storm at landfall.^34^ Power outages during Hurricane Ike impacted almost 4 million customers across 9 states, 2.1 million of whom were in Houston. Ike caused disruptions to offshore gas production, however, on a lesser scale than Katrina and Rita in 2005, despite following similar paths, in part due to lasting effects from the 2005 hurricanes and there being fewer operating production platforms in the Gulf.^35,36^ Harvey made landfall near Corpus Christi, Texas, in August 2017 and caused historic levels of rainfall and catastrophic flooding across Greater Houston.^37^ Petrochemical facilities were severely damaged by the hurricane,^38^ and more than a quarter of the Superfund sites in the area, numerous sewage treatment plants, and hundreds of industrial facilities were affected by flooding and/or incurred possible damage.^39,40^ Majority Hispanic neighborhoods disproportionately experienced excess contaminant releases from petrochemical facilities,^41^ and low SES areas experienced a higher likelihood of releases from toxic sites associated with the storm.^42^

The literature on hurricane-related contaminant releases has expanded over the last two decades as these events have become more common, increasing 15-fold in the period from 2005 to 2008 compared to trends beginning 1990, according to a national assessment of contaminant release reports.^43^ Most research on contaminant releases during the 2005 and 2008 hurricane seasons has focused on impacts to offshore infrastructure, and studies have largely been qualitative and descriptive,^31,32,36^ lacking assessment of geographic proximity of incidents to communities and potential human exposures. Prior studies of Hurricane Harvey^27,41,42^ have considered exposure risks and environmental justice implications of contaminant releases; however, they have focused primarily on air emissions events. Our analysis expands on this body of prior work by examining the magnitude and causes of excess air emissions events from industrial facilities and contaminant releases (e.g., spills) to land and water across a total of 38 impacted counties during Hurricanes Rita, Ike, and Harvey. Unanticipated, short-duration (<24 h) excess air emissions events due to plant start-ups, shutdowns, maintenance, malfunctions, and flaring are regular occurrences and can result in air pollutant emissions that are orders of magnitude higher than during routine operations.^44^ We therefore compare excess contaminant releases during the hurricanes to those reported during reference periods in the year before and after each hurricane in order to distinguish hurricane-attributable releases from business-as-usual events. We then combine information about contaminant release locations and population sociodemographic composition to test the hypothesis that people of color were more likely to live near hurricane-related release events.

## Materials and Methods

We extracted reports of excess air emissions events from the Texas Commission on Environmental Quality’s (TCEQ) database of Air Emissions and Maintenance Events (AEME) and contaminant releases to land and water from the U.S. Coast Guard’s National Response Center’s (NRC) Incident Reporting Information System. We considered time periods that began 2 days prior to each hurricane’s landfall in Texas to account for releases associated with planned facility shutdowns and ended 1 week after each hurricane dissipated or its track was no longer present in Texas, resulting in an 11-day period for Hurricanes Rita and Ike and a 14-day period for Hurricane Harvey. We compared the number of release events during these hurricane periods to excess contaminant releases reported during reference periods of similar dates, as well as randomly sampled days in the year prior to and after each storm. We then conducted separate block group level analyses to estimate the likelihood of residential proximity to an air emissions event or contaminant release to land or water during the hurricane. We grouped Hurricanes Rita and Ike due to their temporal proximity and comparable degree of severity and analyzed Hurricane Harvey alone. We used multivariable regression models to estimate associations between racial/ethnic composition and the likelihood of experiencing an air emissions event and a contaminant release to land or water, controlling for the following covariates: poverty, housing tenure, vehicle ownership, and population density.

### Study Area and Period

We considered hurricanes from 2000 to 2020 classified as Category 2 or higher while making landfall or passing through Texas. We restricted to ones with paths that came within 200 km of Harris County, Texas. Hurricanes Rita (2005), Ike (2008), and Harvey (2017) met these criteria. We defined the study periods for Rita, Ike, and Harvey as follows: September 22–October 2, 2005; September 11–September 21, 2008; and August 23–September 5, 2017, respectively.

**Figure 1:**
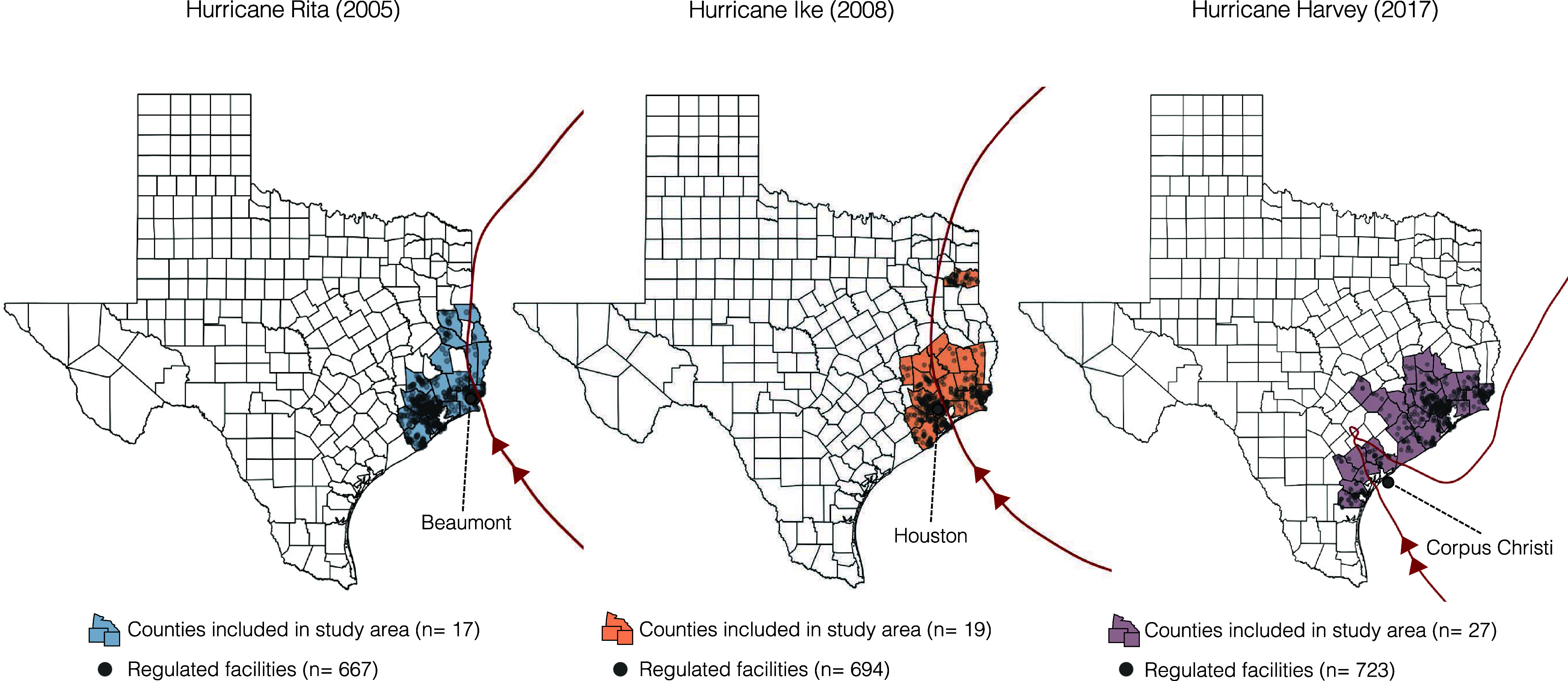
Texas counties with regulated industrial facilities severely impacted by Hurricanes Rita, Ike, and Harvey.

We restricted the study area to a total of 38 unique Texas counties: 17 for Rita, 19 for Ike, and 27 for Harvey (Figure 1). We considered counties that were designated for individual and public assistance by the US Federal Emergency Management Agency (FEMA) and had at least one state-regulated facility that reported to the Point Source Emissions Inventory during the year of each hurricane (i.e., 2005, 2008, and 2017). Next, we restricted to counties that were at risk of a contaminant release due to severe rain or wind impact or the presence of 24 or more regulated facilities, equivalent to the average number of facilities in each FEMA-designated county during all three hurricanes (compared to an average of 8 facilities per county across the entire state and approximately 280 in Harris County.) Counties were considered severely impacted if they experienced either (1) higher than average cumulative rainfall during the hurricane period relative to other FEMA-designated counties (141 mm during Rita, 127 mm during Ike, and 440 mm during Harvey) or (2) peak sustained surface wind at the county’s population mean center ≥64 knots (Category 1 of the Saffir-Simpson Hurricane Wind Scale). We obtained county-level precipitation measures, estimated at the county’s centroid, from the PRISM Climate Group^45^ and data on wind speed and hurricane tracks from the *hurricaneexposure* R package.^46,47^

### Regulated Facilities

We obtained the geographic coordinates and North American Industry Classification System (NAICS) codes for all state-regulated industrial facilities in the 38-county study area from TCEQ’s Point Source Emissions Inventory, an annual survey of industrial sites (e.g., chemical plants, refineries) that meet the reporting criteria described in the TCEQ Emissions Inventory Rule (30 Texas Administrative Code Section 101.10).^48^ This includes facilities emitting at least ten tons per year (tpy) of volatile organic compounds (VOC), 25 tpy of nitrogen oxides, or 100 tpy of any other contaminant subject to the National Ambient Air Quality Standards. We considered facilities that reported to the Point Source Emissions Inventory from 2005 to 2009 and 2016 to 2018. We grouped facilities by NAICS codes into the following categories: petrochemical manufacturing; petroleum refineries; plastics, resin, and other manufacturing; fossil fuel extraction, transmission, and power generation; and warehousing, storage, and other.

### Excess Air Emissions Events

We acquired reports of excess air emissions events from TCEQ’s database of Air Emissions and Maintenance Events (AEME) for 2005 to 2009 and 2016 to 2018.^49^ These include unauthorized or excess releases resulting from accidents (e.g., equipment malfunction) and scheduled or unscheduled maintenance, shutdown, or start-up activities. Facilities are required to report events within 24 h of their occurrence if they exceed an emissions threshold. We restricted to events reported in pounds and combined individually reported pollutants into the following groups: non-methane VOCs, sulfur oxides, carbon monoxide, nitrogen oxides, methane, particulate matter, and other (e.g., carbon dioxide, hydrogen sulfide, and ammonia, among others).

We joined reports of air emissions events with point locations and NAICS codes of regulated facilities from the Point Source Emissions Inventory based on corresponding Regulated Entity Reference Numbers. We classified facilities that reported events based on NAICS codes in matching facility records from the Point Source Emissions Inventory when available. When not available (i.e., did not report to the Point Source Emissions Inventory during the same year as the emissions event), we manually assigned a facility type category based on information available in the AEME data set (e.g., multiple NAICS codes, operator name).

We considered all air emissions events from 2005 to 2009 and 2016 to 2018 that met the time frame and county inclusion criteria in our descriptive analyses. The regression analysis focused more narrowly on events reported from regulated facilities in 2005, 2008, and 2017. Five air emissions events, 1 reported during Rita, 3 during Ike, and 1 during Harvey, were not from a regulated facility and therefore were excluded from the block group level analysis.

### Contaminant Releases to Land or Water

We obtained information on the location, cause, and type of contaminant releases to land and water from the U.S. Coast Guard’s National Response Center’s (NRC) Incident Reporting Information System.^50^ This is a database of field reports for hazardous material releases and spills that have been submitted by the public. Reports include a qualitative description of the incident based on the caller’s testimony, information on the incident’s approximate location, cause, and type, and, in some cases, quantities and classifications of materials released.

We restricted the NRC data to contaminant releases reported to be caused by flood, hurricane, equipment failure, natural phenomenon, sinking vessel, or unknown causes and of the following types: fixed, mobile, pipeline, storage tank, unknown sheen, and vessel. We omitted releases from aircrafts and railroads and releases caused by derailment, dumping, explosion, operator error, overpressuring, and trespassers. To avoid double counting, we removed reports of air emissions releases based on string searches of words such as flaring, scrubber, and atmosphere. We also removed reports of incident status updates (e.g., reports of ongoing incidents), maintaining only the original reports.

We considered all contaminant releases from 2005 to 2009 and 2016 to 2018 that met the inclusion criteria for time frame, county, cause, and type in descriptive analyses. Regression models focused on releases reported in 2005, 2008, and 2017 that could be geocoded. We geocoded reports based on available locational information (e.g., street address and approximate cross streets) using the Google API. A total of 47 reports (7 during Rita, 30 during Ike, and 10 during Harvey) for which we were unable to assign coordinates due to inaccuracies or gaps in the provided locational information were dropped from the block group analysis.

### Demographics and Social Vulnerability Measures

We estimated block group level sociodemographic measures using the U.S. Census Bureau’s American Community Survey’s 2005–2009 5-year estimates and 2000 block group boundaries for our analysis of Hurricanes Rita and Ike, and 2015–2019 5-year estimates and 2017 block group boundaries for our analysis of Harvey. For each block group, we calculated the percentage of the population identified as Hispanic/Latino, Non-Hispanic (NH) Asian, NH Black, NH Native American, NH other race (including multiracial), NH Pacific Islander, and NH White. Due to small sample sizes, we combined NH Native American, NH other race, and NH multiracial. We also combined NH Asian and NH Pacific Islander in our statistical analysis. We constructed the following block group level social vulnerability indicators, which may be associated with hurricane exposure and one’s ability to protect and recover from extreme weather: housing tenure (percentage of renters), vehicle ownership (percentage of households without a vehicle), and poverty (percentage of the population with income below twice the federal poverty level). We also estimated block group population density, expressed as population (100 people) per square kilometer of land area, because communities of color and industrialized areas are more densely populated on average.^51,52^

### Wind Direction

We approximated wind direction for each air emissions event following an approach similar to Cushing et al.^53^ We obtained hourly wind direction from the North American Land Data Assimilation System available in 0.125° grid spacing.^54^ We used zonal (*u*) and meridional (*v*) winds to calculate the absolute wind speed (ws) by taking the square root of the sum of *u* and *v* (√*u*^2^ + *v*^2^ = ws). We then calculated the leeward angle in radians—the direction the wind is blowing toward—using the absolute wind speed and zonal and meridional wind components: *a*tan2 (*v*/ws, *u*/ws). We converted radians to degrees by multiplying by 180/π. We used the median of daily observations (in degrees) to define the predominant wind direction for each day an air emissions event was reported for point locations at which data were available in our study area. Finally, we assigned air emissions events the wind direction of the nearest point and classified block groups that were within 90° of that direction (45° on each side) as downwind. In the case that a facility reported multiple events across multiple days, we considered wind direction for each day, such that nearby block groups may have been downwind from a facility release on some days but not on others.

### Analytic Approach

We first examined the extent, types, and causes of all excess air emissions events and contaminant releases to land and water in the 17-, 19-, and 27-county study areas reported from September 22–October 2, 2005; September 11–21, 2008; and August 23–September 5, 2017; respectively, compared to reports during reference periods of similar dates, with the same number of weekdays and weekends, in the single years prior to (2004, 2007, 2016) and after (2006, 2009, 2018) each hurricane. Reference days were conceived of as business-as-usual periods during which excess contaminant releases to air, water, and land occur in the absence of a hurricane due to a variety of other causes such as accidents, power outages, or maintenance activities. In a secondary analysis, we compared hurricane-period contaminant releases to ones reported on randomly selected days in the single years prior to and after each hurricane. We selected the same number of random days as each hurricane period, excluded reference period dates from the sample pool, and did not consider the day of the week. We considered air emissions events and contaminant releases to water/land in separate analyses due to differences in their severity, sources, reporting mechanisms, and data availability (e.g., more complete data on individual contaminants in the TCEQ data set).

**Figure 2:**
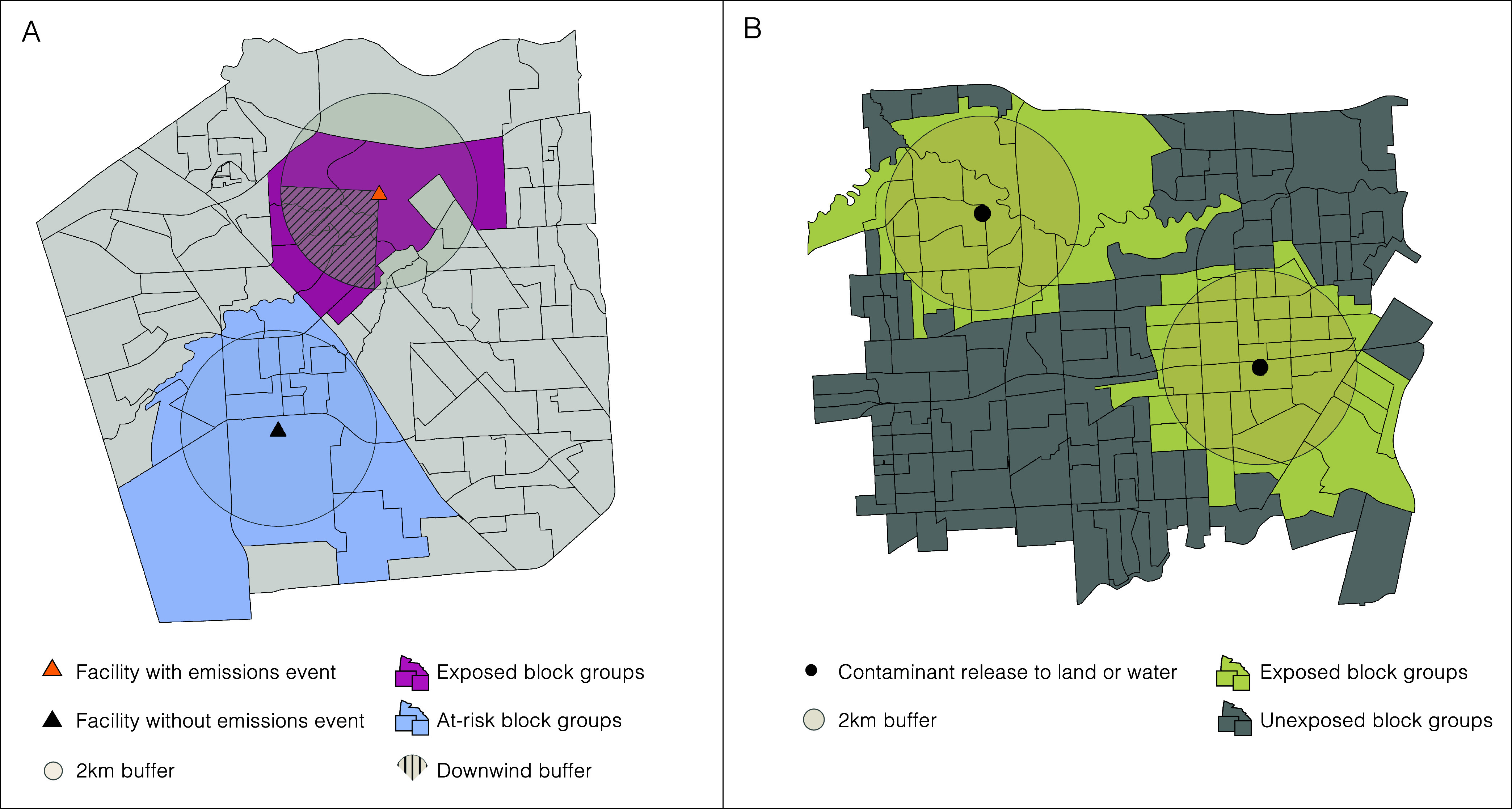
Block group exposure assignment based on proximity to (A) regulated facilities and air emissions events and (B) contaminant releases to land or water. Note. Block groups <2 km and upwind of an air emissions event and ones >10 km of a contaminant release to air, land, or water were excluded.

In our block group level analyses, we combined contaminant releases reported and areas impacted during Hurricanes Rita and Ike and analyzed Hurricane Harvey alone. In our analysis of excess air emissions events, we classified block groups based on their proximity to regulated facilities that reported an air emissions event as follows: (1) exposed: <2 km and downwind of a regulated facility that reported an air emissions event during Hurricanes Rita/Ike or Harvey (excluding block groups <2 km and upwind of an event); (2) at-risk: <2 km from a regulated facility that did not report an event; and (3) unexposed: 2–10 km from a regulated facility, regardless of whether they reported an event during the hurricanes (Figure 2A). We considered the second group of “at-risk” block groups as the primary comparison group since block groups without a regulated facility are not at risk of an excess air emissions event. In a sensitivity analysis of excess air emissions events, we expanded the definition of exposed block groups to include all of those within 2 km of an event, not accounting for wind direction. With respect to contaminant releases to land or water, block groups were classified as either (1) exposed: <2 km of a reported contaminant release or (2) unexposed: 2–10 km of a reported contaminant release (Figure 2B).

We calculated descriptive statistics and correlation coefficients between racial/ethnic variables, social vulnerability indicators, and our outcomes to examine the distribution and bivariate associations among all variables of interest. We then used multivariable regression models to estimate associations between race/ethnicity and two outcomes for Hurricanes Rita and Ike combined and Harvey alone: (1) exposure to an air emissions event (with the reference group being at-risk block groups) and (2) exposure to a contaminant release to land or water (with the reference group being unexposed block groups). Adjusted models controlled for social vulnerability indicators and population density.

We fit logistic regression models for both outcomes and included a fixed effect for counties; however, residuals were spatially autocorrelated based on Moran’s I, violating model assumptions of independent observations. To address spatial autocorrelation, we fit multivariable binomial Leroux conditional autoregressive (CAR) models implemented in a Bayesian setting with Markov chain Monte Carlo (MCMC) simulation using the *CARBayes* package in R (version 6.1.1).^55^ CAR models are commonly used to model nonoverlapping spatial areal data, which typically exhibit spatial autocorrelation. We first created a neighborhood matrix and specified spatial adjacency using a queen criterion of contiguity (i.e., neighbors share a common edge or vertex). Block groups that did not share a common boundary were dropped from the analysis (4 and 2 at-risk block groups in the Rita/Ike and Harvey air emission event models, respectively). Inference for the models was based on 3 MCMC chains running in parallel on 3 cores for 20 000 samples, the first 10 000 of which were removed as the burn-in period. These samples were deemed adequate to offer reliable posterior inference based upon MCMC convergence diagnostics available in the *CARBayes* package as well as based on visual inspection of traceplots. The posterior median estimates and 95% credible intervals (CI) of the odds ratios (OR) are based on their respective posterior distributions. We exponentiated the posterior samples of all fixed effects to obtain the posterior distribution of odds ratios. CAR models are included as the main analysis, and logistic regression models are included as Supporting Information for reference (Table S3).

Unadjusted associations were assessed in models, including the following independent variables, with % NH White as the reference group: % Hispanic, % Black, % Asian/Pacific Islander, and % other races (including Native American and multiracial). Adjusted models included additional social vulnerability indicators chosen a priori: vehicle ownership, poverty, housing tenure, and population density.

## Results

**Figure 3:**
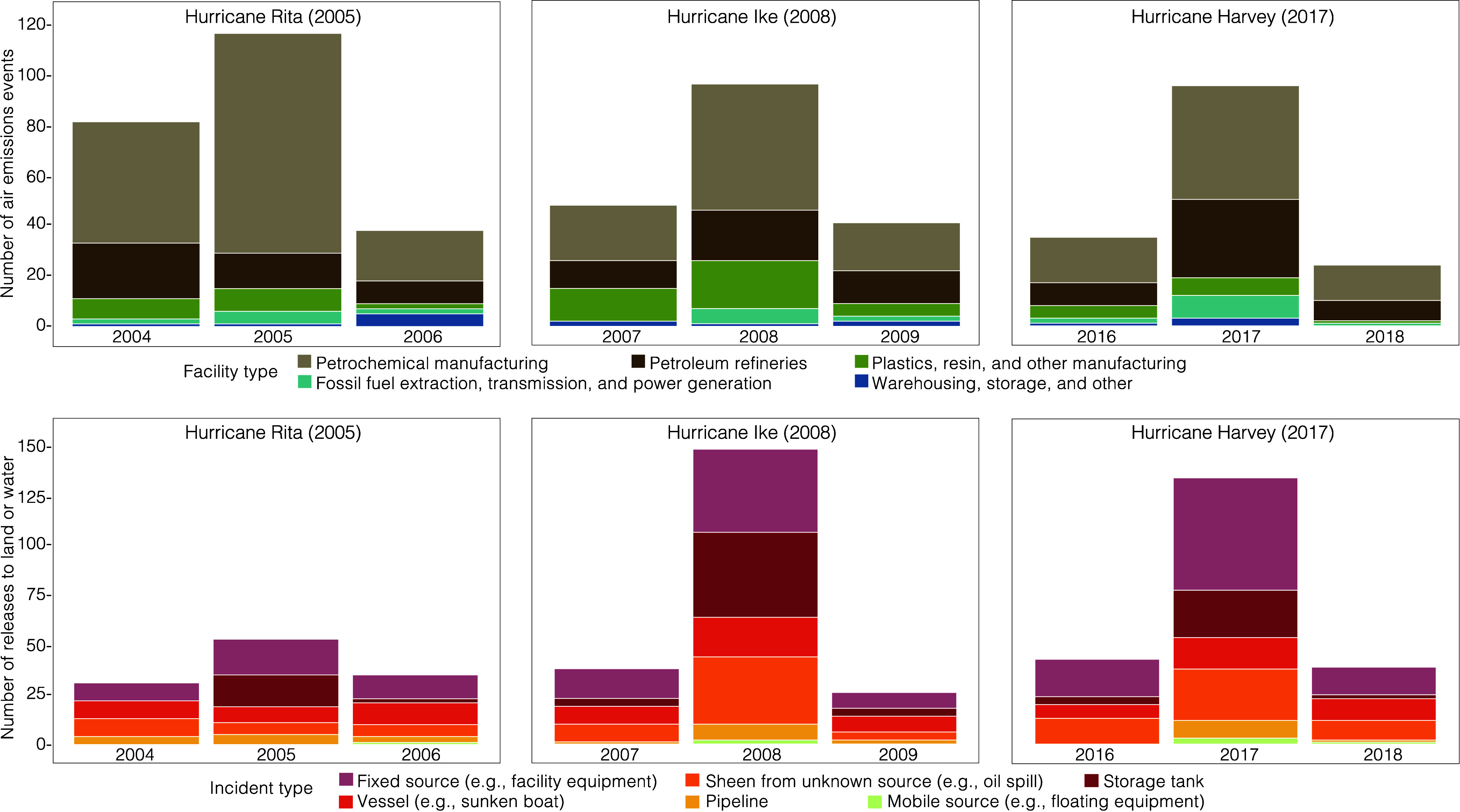
Excess contaminant releases to air and land or water by source category reported during Hurricanes Rita, Ike, and Harvey compared to reference periods. Note. Reference periods in 2004 (September 23–October 3) and 2006 (September 22–October 2); 2007 (September 13–September 23) and 2009 (September 11–September 21); and 2016 (August 23–September 5) and 2018 (August 23–September 5) include similar dates adjusted to include the same number of weekdays and weekends as the Hurricane Rita (September 22–October 2, 2005), Ike (September 11–September 21, 2008), and Harvey (August 23–September 5, 2017) periods, respectively. Contaminant releases to land or water for all years were restricted to those with the following causes: flood, hurricane, natural phenomenon, equipment failure, sinking vessel, or unknown causes.

During the 1.5-week Hurricane Rita period, we estimated 116 reports of excess air emissions events (Figure 3 and Table S1), totaling to more than 4.5 million pounds (Figure S2), compared to an average of 60 events and 259 000 excess pounds during reference periods. We also estimated 53 contaminant releases to land or water during Rita, more than 1.5 times as many than what was reported during reference periods (Figure 3 and Table S1). We estimated 96 excess air emissions events during the 1.5-week Hurricane Ike period (Figure 3 and Table S1), totaling to more than 3 million pounds (Figure S2), compared to an average of 45 events and 614 000 pounds in reference periods. We also estimated 149 contaminant releases to land or water during Ike, almost 5 times more than what was reported on average during reference periods (Figure 3 and Table S1). Finally, we estimated 95 air emissions events during the 2-week Hurricane Harvey period (Figure 3 and Table S1), totaling to more than 10 million pounds (Figure S2), compared to an average of 30 events and 910 000 excess pounds reported in reference years, as well as 135 contaminant releases to land and water, 3 times more than what was reported on average during reference periods (Figure 3 and Table S1). Compared to reference years, the greatest proportion of air emissions events during Rita and Ike were reported from petrochemical manufacturing facilities (75 and 52%, respectively), and petroleum refineries (33%) were responsible for a greater or equal proportion of air emissions events during Harvey, compared to reference years (Figure 3 and Table S1). Storage tanks contributed to a greater proportion of releases to land and water during all three hurricanes compared to reference periods, with fixed sources also contributing to a large proportion (Figure 3 and Table S1). Results from our secondary analysis of hurricane-period contaminant releases compared to those reported during random days showed similar patterns (Figures S1 and S2).

Among the regulated facilities that reported an air emissions event during Rita (*n* = 42), Ike (*n* = 51), and Harvey (*n* = 48), petrochemical manufacturing sites were responsible for the greatest number of events (*n* = 87, 50, and 45, respectively), releasing approximately 2.2, 1.2, and 1.6 million pounds of air contaminants, respectively (Table 1). Petroleum refineries were responsible for the second-greatest number of air emissions events and the greatest quantity (pounds), relative to other facility types during each hurricane, with more than 5.4 million pounds released during Harvey (Table 1). Non-methane VOCs (e.g., isopentane, butane), sulfur oxides, and carbon monoxide were the top air contaminants released by mass (Table 1). Of the contaminant releases to land or water that were geocoded (included in block group analysis), the greatest proportion during Rita and Harvey was from fixed sources (35 and 42%, respectively); storage tanks were responsible for the greatest proportion during Ike (32%) (data not shown). Many of these reports described discharges of oil into water bodies (e.g., rivers, ship channels) or onto land but often listed their specific causes as unknown (e.g., “unspecified oil sheen”).

**Table 1:** Excess Air Emissions Events Reported from Regulated Facilities during Hurricanes Rita, Ike, and Harvey^a^

	Hurricane Rita (*n* = 115)	Hurricane Ike (*n* = 93)	Hurricane Harvey (*n* = 94)
Reported Cause of Air Emissions Events, Count (%)			
start-up	46 (40)	19 (20)	16 (17)
shutdown	24 (21)	27 (29)	10 (11)
maintenance	0 (0)	5 (5)	1 (1)
other	45 (39)	42 (45)	67 (71)
Number of Air Emissions Events Per Facility, Mean (min, max)			
	2.7 (1, 14)	1.8 (1, 8)	2 (1, 5)
Pounds of Air Emissions Released Per Event, Mean (min, max)			
	39 910 (<1, 2 006 126)	33 220 (<1, 674 744)	107 967 (<1, 4 168 882)
Air Emissions Released Per Facility Type, lbs. (*N* Events)^b^			
petroleum refineries	2 364 350 (14)	1 841 154 (20)	5 404 634 (31)
petrochemical manufacturing	2 195 730 (87)	1 208 417 (50)	1 619 514 (45)
plastics, resin, and other manufacturing	15 093 (8)	21 576 (17)	102 267 (7)
fossil fuel extraction, transmission, and power generation	14 274 (5)	18 293 (6)	383 995 (8)
warehousing, storage, and others	187 (1)		2,638,461 (3)
total, lbs.	4 589 634	3 089 440	10 148 871
Air Emissions Released by Contaminant Group, lbs. (*N* Events)^b^^,^^c^			
carbon monoxide	1 401 615 (55)	709 918 (56)	1 899 263 (57)
sulfur oxides	1 196 592 (7)	1 097 131 (17)	2 414 759 (27)
methane	1 027 729 (11)	27 519 (10)	477 844 (12)
non-methane volatile organic compounds	803 798 (65)	999 686 (83)	4 281 830 (85)
nitrogen oxides	119 180 (58)	115 057 (56)	935 869 (56)
other^d^	39 616 (16)	62 644 (35)	69 535 (38)
particulate matter	1103 (3)	77 487 (5)	69 770 (14)

a Descriptive statistics are based on excess air emissions events reported from regulated facilities that were reported to the Point Source Emissions Inventory in 2005, 2008, and 2017.

b Total pounds released by facility type and contaminant group may not be equal due to rounding.

c The numbers of air emissions events do not add up to 115, 93, and 94, respectively, because individual contaminants may have been released multiple times during different reported events.

d The “other” group includes carbon dioxide, hydrogen, hydrogen sulfide, ammonia, acetone, and lead, among other contaminants.

We found that 74 block groups were exposed (<2 km and downwind) to at least one air emissions event from a regulated facility, and 285 were exposed (<2 km) to at least one contaminant release to land or water during Rita. On average, block groups exposed to air emissions events during Rita had higher mean percentages of Hispanic and White residents and lower percentages of Black and Asian residents compared to at-risk or unexposed block groups. Block groups exposed to contaminant releases to land or water had higher percentages of White residents, renters, and households without a vehicle compared to unexposed block groups (Table 2).

**Table 2:** Sociodemographic Characteristics in Block Groups Exposed, Unexposed, and at Risk of Exposure to Contaminant Releases during Hurricanes Rita, Ike, and Harvey^a^

	air emissions events									releases to land and water					
	Hurricane Rita^b^			Hurricane Ike^c^			Hurricane Harvey^d^			Hurricane Rita^e^		Hurricane Ike^f^		Hurricane Harvey^g^	
sociodemographics, mean	exposed (*n* = 74)	at-risk (*n* = 1428)	unexposed (*n* = 1561)	exposed (*n* = 86)	at-risk (*n* = 1397)	unexposed (*n* = 1649)	exposed (*n* = 116)	at-risk (*n* = 1331)	unexposed (*n* = 2, 237)	exposed (*n* = 285)	unexposed (*n* = 1684)	exposed (*n* = 509)	unexposed (*n* = 1801)	exposed (*n* = 639)	unexposed (*n* = 2293)
% hispanic	34.3	33.5	29.4	31.5	32.8	28.5	47.5	39.3	38.9	32.6	34.3	32.0	31.6	39.6	40.8
% white	48.5	41.5	46.2	44.9	42.0	47.2	32.6	36.5	38.5	45.9	38.7	46.4	42.4	40.5	33.8
% black	13.9	20.0	18.7	20.0	20.2	18.7	17.4	17.8	15.3	16.1	21.0	16.9	20.6	14.3	18.4
% asian	2.1	3.7	4.3	2.3	3.7	4.1	1.1	4.7	5.4	3.9	4.8	3.4	4.0	3.9	5.1
% other^h^	1.2	1.2	1.4	1.2	1.3	1.4	1.4	1.7	1.8	1.4	1.2	1.3	1.3	1.6	1.8
% Pacific Islander	0.10	0.05	0.07	0.08	0.04	0.07	0.06	0.04	0.05	0.10	0.04	0.06	0.07	0.03	0.05
% poverty^i^	40.2	41.9	36.7	41.8	41.6	36.4	43.0	38.5	35.1	40.8	41.0	41.1	38.9	38.7	37.8
% renters	30.5	38.6	33.6	31.1	38.4	33.6	38.9	40	38.1	41.6	39.4	43.5	37.3	41.0	41.6
% without a vehicle	7.3	8.7	6.6	6.8	8.7	6.5	9.6	6.7	6.0	9.7	8.7	9.9	7.9	7.3	6.7
population density (100 people per km^2^), mean	7.6	15.6	17.7	7.0	15.4	17.3	7.9	16.8	21.0	16.3	19.8	17.3	18.7	16.9	22.4

a We classified block groups based on their proximity to regulated facilities that reported an air emissions event as follows: (1) exposed: <2 km and downwind of a regulated facility that reported an air emissions event; (2) at-risk: <2 km from a regulated facility that did not report an event; and (3) unexposed: 2–10 km from a regulated facility. With respect to contaminant releases to land or water, block groups were classified as either (1) exposed: < 2 km of a reported contaminant release or (2) unexposed: 2–10 km of a reported contaminant release.

b Based on 115 emissions events reported from regulated facilities in 17 Texas counties from 9/22 to 10/2/2005, 2000 BG boundaries, and demographic data from ACS 2005–2009.

c Based on 93 emissions events reported from regulated facilities in 19 Texas counties from 9/11 to 9/21/2008, 2000 BG boundaries, and demographic data from ACS 2005–2009.

d Based on 94 emissions events reported from regulated facilities in 27 Texas counties from 8/23 to 9/5/2017, 2017 BG boundaries, and demographic data from ACS 2015–2019.

e Based on 46 contaminant releases to land or water reported in 17 Texas counties from 9/22 to 10/2/2005, 2000 BG boundaries, and demographic data from ACS 2005–2009.

f Based on 119 contaminant releases to land or water reported in 19 Texas counties from 9/11 to 9/21/2008, 2000 BG boundaries, and demographic data from ACS 2005–2009.

g Based on 125 contaminant releases to land or water reported in 27 Texas counties from 8/23 to 9/5/2017, 2017 BG boundaries, and demographic data from ACS 2015–2019.

h The “other” group includes non-Hispanic (NH) Native American, NH other race, and NH multiracial.

i Defined as a population with income below twice the federal poverty level.

During Hurricane Ike, 86 block groups were exposed to at least one air emissions event from a regulated facility, and 509 were exposed to at least one contaminant release to land or water. Mean percentages of Hispanic and Black residents as well as renters and households without a vehicle were highest in at-risk block groups, while exposed block groups had higher percentages of White residents and poverty on average. Block groups exposed to contaminant releases to land or water had higher percentages of Hispanic and White residents, poverty, renters, and households without a vehicle, compared to unexposed block groups (Table 2). During Harvey, 116 block groups were exposed to at least one air emissions event from a regulated facility, and 639 were exposed to at least one contaminant release to land or water. On average, block groups exposed to air emissions events had higher mean percentages of Hispanic residents and households in poverty and without a vehicle compared to at-risk or unexposed block groups. Block groups exposed to contaminant releases to land or water had higher percentages of White residents and households in poverty and without a vehicle than unexposed block groups (Table 2). Block groups exposed to contaminant releases to air, land, or water during any of the 3 hurricanes also had lower average population density, compared to at-risk or unexposed block groups.

The fully adjusted CAR model of air emissions events during Rita and Ike combined showed a 5% higher likelihood of exposure per one-percent increase in Hispanic residents (OR [95% CI] = 1.05 [1.00, 1.13]; Table 3) as well as a 1, 2, 5, and 31% higher likelihood per percent increase in poverty, Black and Asian/Pacific Islander residents, and other people of color, respectively, although credible intervals were wide (OR [95% CI] = 1.01 [0.95, 1.07]; 1.02 [0.98, 1.08]; 1.05 [0.87, 1.21]; 1.31 [0.86, 1.76]; Table 3). The fully adjusted CAR model of air emissions events during Harvey showed a 10% increase in the likelihood of exposure per percent increase in Hispanic residents (OR [95% CI] = 1.10 [1.00, 1.23]; Table 3). We also found that a one-percent increase in Asian/Pacific Islander and Black residents and other people of color, as well as renters and households without a vehicle, was associated with a 1, 5, 21, 3, and 18% higher likelihood, respectively, of being exposed to an air emissions event during Harvey, but the estimates were less precise (OR [95% CI] = 1.01 [0.66, 1.57]; 1.05 [0.94, 1.18]; 1.21 [0.60, 2.28]; 1.03 [0.94, 1.13]; 1.18 [0.96, 1.47]; Table 3). Population density was associated with a decrease in the likelihood of exposure to an air release event during all hurricanes (OR [95% CI] = 0.64 [0.52, 0.73] for Rita and Ike; 0.46 [0.31, 0.62] for Harvey; Table 3). Effect estimates were consistent in direction and, in some cases, stronger in our sensitivity analysis that did not account for wind direction to define exposure, including for % Hispanic in the adjusted CAR model for Rita and Ike (OR [95% CI] = 1.06 [1.02, 1.12]) and other people of color in the adjusted model for Harvey (1.51 [1.00, 2.42]; Table S2).

**Table 3:** Association between Neighborhood Sociodemographic Characteristics and Risk of Exposure to a Contaminant Release to Air and Land or Water during Hurricanes Rita, Ike, and Harvey (Odds Ratios and 95% Credible Intervals from Conditional Autoregressive Models)^a^

	air emissions events				releases to land or water			
	Hurricanes Rita and Ike^b^ exposed (*n* = 119) vs at-risk BGs (*n* = 1494)		Hurricane Harvey^c^ exposed (*n* = 116) vs at-risk BGs (*n* = 1331)		Hurricanes Rita and Ike^d^ exposed (*n* = 638) vs unexposed BGs (*n* = 1937)		Hurricane Harvey^e^ exposed (*n* = 639) vs unexposed BGs (*n* = 2293)	
	unadjusted	adjusted	unadjusted	adjusted	unadjusted	adjusted	unadjusted	adjusted
% hispanic	1.02 (0.97, 1.06)	1.05 (1.00, 1.13)	1.01 (0.94, 1.08)	1.10 (1.00, 1.23)	0.99 (0.95, 1.03)	1.00 (0.94, 1.05)	0.97 (0.93, 1.01)	0.97 (0.92, 1.02)
% black	1.01 (0.96, 1.04)	1.02 (0.98, 1.08)	1.03 (0.96, 1.12)	1.05 (0.94, 1.18)	0.98 (0.94, 1.02)	0.96 (0.92, 1.01)	0.97 (0.93, 1.02)	0.94 (0.89, 1.00)
% Asian/Pacific Islander	1.09 (0.94, 1.24)	1.05 (0.87, 1.21)	0.92 (0.63, 1.26)	1.01 (0.66, 1.57)	1.09 (0.98, 1.21)	1.09 (0.98, 1.22)	1.02 (0.92, 1.13)	1.03 (0.91, 1.15)
% other	1.02 (0.76, 1.63)	1.31 (0.86, 1.76)	1.19 (0.78, 1.99)	1.21 (0.60, 2.28)	1.11 (0.87, 1.45)	1.04 (0.79, 1.36)	0.86 (0.68, 1.09)	0.83 (0.64, 1.06)
% without a vehicle		0.95 (0.79, 1.04)		1.18 (0.96, 1.47)		1.01 (0.93, 1.09)		1.06 (0.96, 1.17)
% poverty		1.01 (0.95, 1.07)		0.96 (0.86, 1.08)		0.98 (0.93, 1.04)		1.06 (1.01, 1.12)
% renters		0.97 (0.94, 1.03)		1.03 (0.94, 1.13)		1.07 (1.03, 1.11)		1.02 (0.98, 1.06)
population density (100 people per km^2^)		0.64 (0.52, 0.73)		0.46 (0.31, 0.62)		0.85 (0.79, 0.90)		0.86 (0.80, 0.91)

a BGs = block groups.

b Based on a total of 208 emissions events reported from regulated facilities, 2000 BG boundaries, and demographic data from ACS 2005–2009.

c Based on 94 emissions events reported from regulated facilities, 2017 BG boundaries, and demographic data from ACS 2015–2019.

d Based on a total of 165 contaminant releases to land or water, 2000 BG boundaries, and demographic data from ACS 2005–2009.

e Based on 125 contaminant releases to land or water, 2017 BG boundaries, and demographic data from ACS 2015–2019.

The fully adjusted CAR model examining exposure to contaminant releases to land and water during Hurricanes Rita and Ike combined showed that a one-percent increase in renters was associated with a 7% higher likelihood of exposure (OR [95% CI] = 1.07 [1.03, 1.11]; Table 3). A one-percent increase in Asian/Pacific Islander residents, other people of color, and households without a vehicle was associated with a 9, 4, and 1% higher likelihood, respectively, of being exposed, but the estimates were less precise (OR [95% CI] = 1.09 [0.98, 1.22]; 1.04 [0.79, 1.36]; 1.01 [0.93, 1.09]; Table 3). The fully adjusted CAR model for Hurricane Harvey showed that a one-percent increase in poverty was associated with a 6% increase in the likelihood of exposure (1.06 [1.01, 1.12]; Table 3) and a percent increase in Black residents was associated with a slightly lower likelihood (OR [95% CI] = 0.94 [0.89, 1.00]; Table 3). Households without a vehicle and renters were also associated with an increase in the likelihood of a contaminant release to land or water, although estimates were less precise (OR [95% CI] = 1.06 [0.96, 1.17]; 1.02 [0.98, 1.06]; Table 3). Population density was associated with a decreased likelihood across all hurricanes (OR [95% CI] = 0.85 [0.79, 0.90] for Rita and Ike; 0.86 [0.80, 0.91] for Harvey; Table 3).

## Discussion

In our examination of self-reported contaminant releases to air, water, and land, we found that, on average, Hurricane Rita resulted in approximately twice as many releases than reported during reference periods during the years post and prior, and Hurricanes Ike and Harvey resulted in more than three times as many compared to reference years. Regulated petrochemical manufacturing facilities accounted for the greatest number of excess air emissions events during Rita, Ike, and Harvey, and refineries released the greatest quantity (in pounds) relative to other facility types for each hurricane. This is in line with prior research by Flores et al. analyzing air emissions events from TCEQ’s database due to Hurricane Harvey from 42 petrochemical facilities, including manufacturing and gas processing sites, refineries, and chemical terminals across 12 Gulf Coast counties from August 24–31, 2017, releasing an average of almost 140 000 pounds each.^41^ Prior work characterizing hazardous substance release events from industrial settings attributable to Hurricanes Katrina and Rita (*n* = 166 events, 79% of which occurred in Texas and 21% in Louisiana) also found that a majority were from chemical manufacturing (69%) and petroleum and coal manufacturing (20%) sites, with the remaining events originating from the mining, utilities, and construction industries.^17^ These release events were reported to the Hazardous Substances Emergency Events Surveillance system maintained by the Agency for Toxic Substances and Disease Registry.

We also found that large proportions of releases to land and water originated from fixed sources (34, 28, and 42% for Rita, Ike, and Harvey, respectively) and storage tanks (30, 29, and 18% for Rita, Ike, and Harvey, respectively). These findings align with conclusions from a national assessment by Sengul et al. of contaminant releases reported to the NRC from 1990 to 2008 documenting that the largest proportion of overall releases originated from fixed facilities (26%) and 11% from storage tanks.^43^ The majority of releases from these sources resulted from hurricanes, floods, and rain. Similarly, prior studies by Qin et al. and Misuri et al. document large proportions of contaminant releases during Harvey from storage equipment across various industry types.^38,56^ These studies highlight the structural vulnerability of fixed sources, as well as atmospheric chemical storage tanks, petroleum storage tanks, and external floating roof tanks, to extreme rainfall and flooding.

Non-methane VOCs, sulfur oxides, and carbon monoxide were the top air pollutants released in excess from regulated facilities, according to reports. A prior assessment of excess air emissions events reported to TCEQ from 2004 to 2015 across Texas similarly found that these pollutants were the most commonly released.^57^ They document 104 202 excess tons of VOCs emitted during this combined period, which is equivalent to 7.5% of total routine emissions from regulated facilities reporting to the Point Source Emissions Inventory.

In our analysis of Hurricane Harvey, neighborhoods with higher percentages of Hispanic residents were more likely to be located near and downwind of Harvey-related air emissions events. This held true even when comparing only among neighborhoods near regulated facilities subject to reporting requirements (exposed versus at-risk block groups), suggesting racial/ethnic disparities in exposure risk to excess air emission events beyond the underlying elevated risk of living near an industrial facility. Our finding is consistent with prior work showing neighborhoods with higher proportions of Hispanic residents had greater densities of petrochemical facilities reporting Harvey-related emissions releases.^41^ In that analysis, the authors defined exposure using a petrochemical hazard density index, which assigns a hazard score to census tracts based on the density of petrochemical sites that had Harvey-related releases within a 1 km buffer. Our block group level study considered a 2 km buffer as well as wind direction in defining exposed block groups. We also included a broader geographic scope and larger number of industries than this prior analysis and controlled for the presence of regulated facilities.

Similar to Harvey, findings from our analysis of Rita and Ike suggested an association between race/ethnicity and the likelihood of living in close proximity to an air emissions event, particularly for neighborhoods with higher percentages of Hispanic residents. Our findings also suggested an increase in the likelihood of exposure associated with higher percentages of Black and Asian/Pacific Islander residents and other people of color, including Native Americans, although these estimates were less precise. These findings are somewhat consistent with those from a study by Li et al. that examined the likelihood of all-cause excess air emissions events in Texas from 2000 to 2010 and found a positive association between excess emissions and the percentage of Black population.^58^ Inconsistencies in findings are likely due to differences in the study design, with our study focusing exclusively on hurricane-related releases in hurricane-affected counties. We also note that differences in our findings between Hurricanes Rita and Ike versus Harvey may have to do with differences in geographic areas affected by each storm (Figure 1), the unusual severity of flooding during Harvey in contrast to the other storms, and changing demographics over the 12-year span we considered.

A contrasting finding from our analysis is that, on average, neighborhoods with higher proportions of people of color were less likely to be located near reports of hazardous substance releases to land and water. This was the case for both hurricane models. Instead, an increase in the percentage of renters was associated with releases to land and water in our analysis of Hurricanes Rita and Ike, and an increase in the percentage of poverty was associated with an increase in the likelihood of exposure during Harvey. Socioeconomic disparities in exposure risk were also documented in a study by Lieberman-Cribbin et al. that reported higher odds of toxic release incidents in low SES census tracts compared to higher SES tracts in Greater Houston during Harvey.^42^ They distinguished between high vs low SES tracts based on a score combining estimates of income, poverty, housing characteristics, education, and employment; however, racial/ethnic makeup was not considered. This study combined a broad range of toxic release types from the Sierra Club’s database on toxic release incidents during Hurricane Harvey sourced from multiple data sources, including the US Coast Guard’s NRC, TCEQ, EPA Toxic Release Inventory, and the Energy Information Administration. It is possible that the differences we observed with respect to race and ethnicity were because we were not able to control for the prior presence of potential sources of contaminant releases to land or water as we did in our analysis of air emissions events. The NRC releases are also self-reported by individual members of the public, which likely results in differential reporting rates that may introduce bias.

Effect estimates from our multivariable models of all hurricanes suggested that a less dense population was associated with a higher risk of all types of contaminant releases nearby, holding race/ethnicity, poverty, and other factors constant, similar to findings by Flores et al.^41^ This is also in line with findings by Li et al., suggesting that a one SD-unit increase in population density (5 persons/acre) is associated with a 96% decrease in the probability of having an excess air emissions event.^58^ This may be the result of poorer routine maintenance or storm-related access to remote equipment and facilities. The disproportionate impact of natech disasters on rural communities may compound existing disparities in access to employment, health care, and other resources, and resulting growing life expectancy gap relative to urban areas.^59−61^

We were limited by the availability of reliable information on pollutant releases, especially with respect to land and water, which likely resulted in underestimates of hurricane-related contaminant releases. We also did not attempt to model exposure to air pollutants, given the complexity of such a task during storm conditions and the unavailability of consistent data from regulatory ambient air pollutant monitoring stations near release events, some of which were offline due to the hurricanes. We instead relied upon proximity and wind direction to identify potentially affected populations. Our outcome measures should therefore be interpreted as indications of potential exposure risk to contaminant releases rather than as measures of exposure or health threat. It is possible that populations farther away from contaminant releases were affected and that the patterns we observed with respect to race/ethnicity and SES would differ with a more precise exposure assessment.

While we did not assess exposure, prior research has suggested evidence of increased contaminant concentrations in the wake of Hurricanes Katrina, Rita, and Harvey. For example, several analyses, including longitudinal studies, have documented increased concentrations of VOCs and polycyclic aromatic hydrocarbons (PAH) in recreational and residential areas and water bodies in Greater Houston after Harvey compared to prehurricane levels.^62−66^ An analysis of drinking water samples from locations adjacent to a superfund site in Beaumont 3 weeks after Hurricane Harvey showed greater than 2 orders of magnitude increase in PAHs due to mobilization of pollutants from flooding.^66^ Additionally, an assessment of sediment cores in the Gulf of Mexico following Hurricanes Katrina and Rita suggested that substantial amounts of prehurricane PAH-enriched sediment derived from offshore petroleum activity were remobilized and redistributed in areas of relatively shallow water.^67^ Some PAHs are known carcinogens, teratogens, and mutagens, and therefore pose serious potential health risks.^68^ They are commonly formed from the incomplete combustion of organic materials like coal and oil, suggesting redistribution of these contaminants from petrochemical sites or power plants due to flooding.

We were not able to control for the quantity of air emissions prior to the hurricane in our analysis; however, after controlling for the prior presence of regulated facilities, our findings suggest racial/ethnic disparities in exposure risks beyond the underlying risks of living near an industrial facility. This conclusion is in line with the findings by Lieberman-Cribbin et al. that similarly documented more hurricane-related incidents at toxic sites in areas of low SES, after accounting for the disproportionate distribution of toxic sites in these areas.^42^ These disparities might exist due to neglect or poor maintenance of infrastructure or lack of preparedness for severe rainfall. For example, reports of air emission events cite leaks from heat exchangers, pipes, or valves and flooding or damage to external floating roof tanks as causes.

In summary, we found disparities in the distribution of excess contaminant releases triggered by Gulf Coast hurricanes with respect to race, housing tenure, income, and rurality. Our findings highlight social inequalities in the risks posed by hydrometeorological natech disasters. Hydrometeorological events are an increasing focus of the natech literature that has traditionally focused on geological hazards such as earthquakes,^69,70^ a testament to the increasing threat of cascading impacts due to extreme weather in the context of climate change. Additional safeguards are needed to prevent hazardous releases and increase climate resilience in fenceline communities.

## Supporting Information Available

Summary table of contaminant releases by source category during hurricane and reference periods; full model results from sensitivity analysis and logistic regression; and bar charts of hurricane-period contaminant releases compared to random days and reference periods (PDF)
