# Supplementary material for: Climate Justice Implications of Natech Disasters: Excess Contaminant Releases during Hurricanes on the Texas Gulf Coast
Source: Environ Sci Technol. 2024 Jul 30;58(32):14180–92. doi: 10.1021/acs.est.3c10797 (PMC11325638; doi:10.1021/acs.est.3c10797)
Supplement: Supplementary file 1 — es3c10797_si_001.pdf [file es3c10797_si_001.pdf]

Supporting Information

Climate Justice Implications of Natech Disasters: Excess Contaminant Releases During Hurricanes on the Texas Gulf Coast

Alique G. Berberian, Rachel Morello-Frosch, Seigi Karasaki, Lara J. Cushing

Summary: 6 pages, 3 tables, 2 figures

Table of Contents

Table S1. Excess contaminant releases to air and land or water by source category reported during Hurricanes Rita, Ike, and Harvey compared to reference periods……… S2

Table S2. Association between neighborhood sociodemographic characteristics and risk of exposure to a contaminant release to air during Hurricanes Rita, Ike, and Harvey (Odds Ratios and 95% Credible Intervals from sensitivity analysis) ….……………….…….. S3

Table S3. Association between neighborhood sociodemographic characteristics and risk of exposure to a contaminant release to air and land or water during Hurricanes Rita, Ike, and Harvey (Odds Ratios and 95% Confidence Intervals from logistic regression)....S4

Figure S1. Excess contaminant releases to air and land or water by source category reported during Hurricanes Rita, Ike, and Harvey compared to random samples in reference years ……………………………………………………………………………… S5

Figure S2. Pounds of excess air contaminants released during Hurricanes Rita, Ike, and Harvey compared to reference periods and random samples …… S6

Table S1. Excess contaminant releases to air and land or water by source category reported during Hurricanes Rita, Ike, and Harvey compared to reference periods

	Rita			Ike			Harvey		
**Source**	2004	**2005**	2006	2007	**2008**	2009	2016	**2017**	2018
**Air emissions events, count (%)**									
Petrochemical manufacturing	48 (59)	**87 (75)**	20 (53)	22 (46)	**50 (52)**	19 (46)	18 (51)	**45 (47)**	14 (58)
Petroleum refineries	22 (27)	**14 (12)**	9 (24)	11 (23)	**20 (21)**	13 (32)	9 (26)	**31 (33)**	8 (33)
Plastics, resin, and other manufacturing	8 (10)	**9 (8)**	2 (5)	13 (27)	**19 (20)**	5 (12)	5 (14)	**7 (7)**	1 (4)
Fossil fuel extraction, transmission, and power generation	2 (2)	**5 (4)**	2 (5)	-	**6 (6)**	2 (5)	2 (6)	**9 (9)**	1 (4)
Warehousing, storage, and other	1 (1)	**1 (<1)**	5 (13)	2 (4)	**1 (1)**	2 (5)	1 (3)	**3 (3)**	-
Total	81	**116**	38	48	**96**	41	35	**95**	24
**Contaminant releases to land or water, count (%)**									
Fixed source (e.g., facility equipment)	9 (29)	**18 (34)**	12 (34)	15 (39)	**42 (28)**	8 (31)	19 (44)	**57 (42)**	14 (36)
Sheen from unknown source (e.g., oil spill)	9 (29)	**6 (11)**	6 (17)	9 (24)	**34 (23)**	4 (15)	13 (30)	**26 (19)**	10 (26)
Vessel (e.g., sunken boat)	9 (29)	**8 (15)**	11 (31)	9 (24)	**20 (13)**	8 (31)	7 (16)	**16 (12)**	11 (28)
Storage tank	-	**16 (30)**	2 (6)	4 (11)	**43 (29)**	4 (15)	4 (9)	**24 (18)**	2 (5)
Pipeline	4 (13)	**5 (9)**	3 (9)	1 (3)	**8 (5)**	2 (8)	-	**9 (7)**	1 (3)
Mobile source (e.g., floating equipment)	-	**-**	1 (3)	-	**2 (1)**	-	-	**3 (2)**	1 (3)
Total	31	**53**	35	38	**149**	26	43	**135**	39

Note. Descriptive statistics are based on raw data and do not consider whether air emission events were from a regulated facility or whether geocoding was possible for contaminant releases to land or water. Total percentages may not equal 100 due to rounding.

Table S2. Association between neighborhood sociodemographic characteristics and risk of exposure to a contaminant release to air during Hurricanes Rita, Ike, and Harvey (Odds Ratios and 95% Credible Intervals from sensitivity analysis)

	**Hurricanes Rita and Ike**[1] Exposed (n=183) vs. At-risk BGs (n=1494)		**Hurricane Harvey**[2] Exposed (n=197) vs. At-risk BGs (n=1331)	
	Unadjusted	Adjusted	Unadjusted	Adjusted
% Hispanic	1.05 (1.00, 1.09)	1.06 (1.02, 1.12)	1.02 (0.97, 1.10)	1.08 (1.00, 1.18)
% Black	1.00 (0.96, 1.04)	1.01 (0.96, 1.05)	1.02 (0.95, 1.10)	1.04 (0.95, 1.14)
% Asian/ Pacific Islander	1.02 (0.89, 1.23)	1.01 (0.87, 1.20)	1.00 (0.81, 1.22)	0.98 (0.72, 1.25)
% Other	0.93 (0.71, 1.31)	0.90 (0.58, 1.42)	1.31 (0.93, 1.98)	1.51 (1.00, 2.42)
% Without a vehicle	-	1.03 (0.96, 1.17)	-	1.07 (0.91, 1.28)
% Poverty	-	1.02 (0.95, 1.08)	-	0.98 (0.90, 1.08)
% Renters	-	0.97 (0.93, 1.00)	-	0.99 (0.92, 1.06)
Population density (100 people per km^2)	-	0.73 (0.70, 0.86)	-	0.73 (0.62, 0.83)

[1] Based on a total of 208 emissions events reported from regulated facilities, 2000 BG boundaries, and demographic data from ACS 2005-2009
[2] Based on a total of 94 emissions events reported from regulated facilities, 2017 BG boundaries, and demographic data from ACS 2015-2019

Note. Exposed block groups are defined as those <2km from an air emissions event, regardless of wind direction

Table S3. Association between neighborhood sociodemographic characteristics and risk of exposure to a contaminant release to air and land or water during Hurricanes Rita, Ike, and Harvey (Odds Ratios and 95% Confidence Intervals from logistic regression)

	Air emissions events				Releases to land or water			
	Hurricanes Rita and Ike		Hurricane Harvey		Hurricanes Rita and Ike		Hurricane Harvey	
	Exposed (n=119) vs. At-risk BGs (n=1494)		Exposed (n=116) vs. At-risk BGs (n=1331)		Exposed (n=638) vs. At-risk BGs (n=1937)		Exposed (n=639) vs. At-risk BGs (n=2293)	
% Hispanic	1.01 (1.00, 1.01)	1.01 (1.00, 1.02)	1.02 (1.01, 1.03)	1.03 (1.01, 1.04)	1.00 (0.99, 1.00)	1.00 (0.99, 1.00)	1.00 (0.99, 1.00)	0.99 (0.99, 1.00)
% Black	1.00 (0.99, 1.00)	1.00 (0.99, 1.01)	1.00 (0.99, 1.01)	0.99 (0.98, 1.01)	0.99 (0.99, 1.00)	0.98 (0.98, 0.99)	0.98 (0.98, 0.99)	0.98 (0.97, 0.98)
% Asian/Pacific Islander	0.97 (0.93, 1.01)	0.98 (0.94, 1.02)	0.91 (0.84, 0.97)	0.93 (0.86, 0.99)	1.00 (0.98, 1.01)	0.99 (0.98, 1.01)	0.98 (0.97, 0.99)	0.98 (0.97, 0.99)
% Other	0.99 (0.89, 1.08)	1.01 (0.91, 1.10)	1.03 (0.94, 1.12)	1.02 (0.93, 1.11)	1.00 (0.95, 1.04)	0.98 (0.94, 1.02)	0.98 (0.95, 1.01)	0.98 (0.94, 1.01)
% Without a vehicle	-	0.99 (0.96, 1.01)	-	1.04 (1.01, 1.06)	-	1.02 (1.00, 1.03)	-	1.01 (1.00, 1.03)
% Poverty	-	1.01 (1.00, 1.03)	-	0.99 (0.98, 1.01)	-	1.00 (0.99, 1.01)	-	1.01 (1.00, 1.01)
% Renters	-	0.99 (0.98, 1.00)	-	1.00 (0.99, 1.02)	-	1.01 (1.01, 1.02)	-	1.01 (1.00, 1.01)
Population density (100 people per km^2)	-	0.94 (0.92, 0.97)	-	0.94 (0.91, 0.96)	-	0.99 (0.98, 0.99)	-	0.99 (0.98, 0.99)

Note. Models include a fixed effect for county.

Figure S1. Excess contaminant releases to air and land or water by source category reported during Hurricanes Rita, Ike, and Harvey compared to random samples in reference years

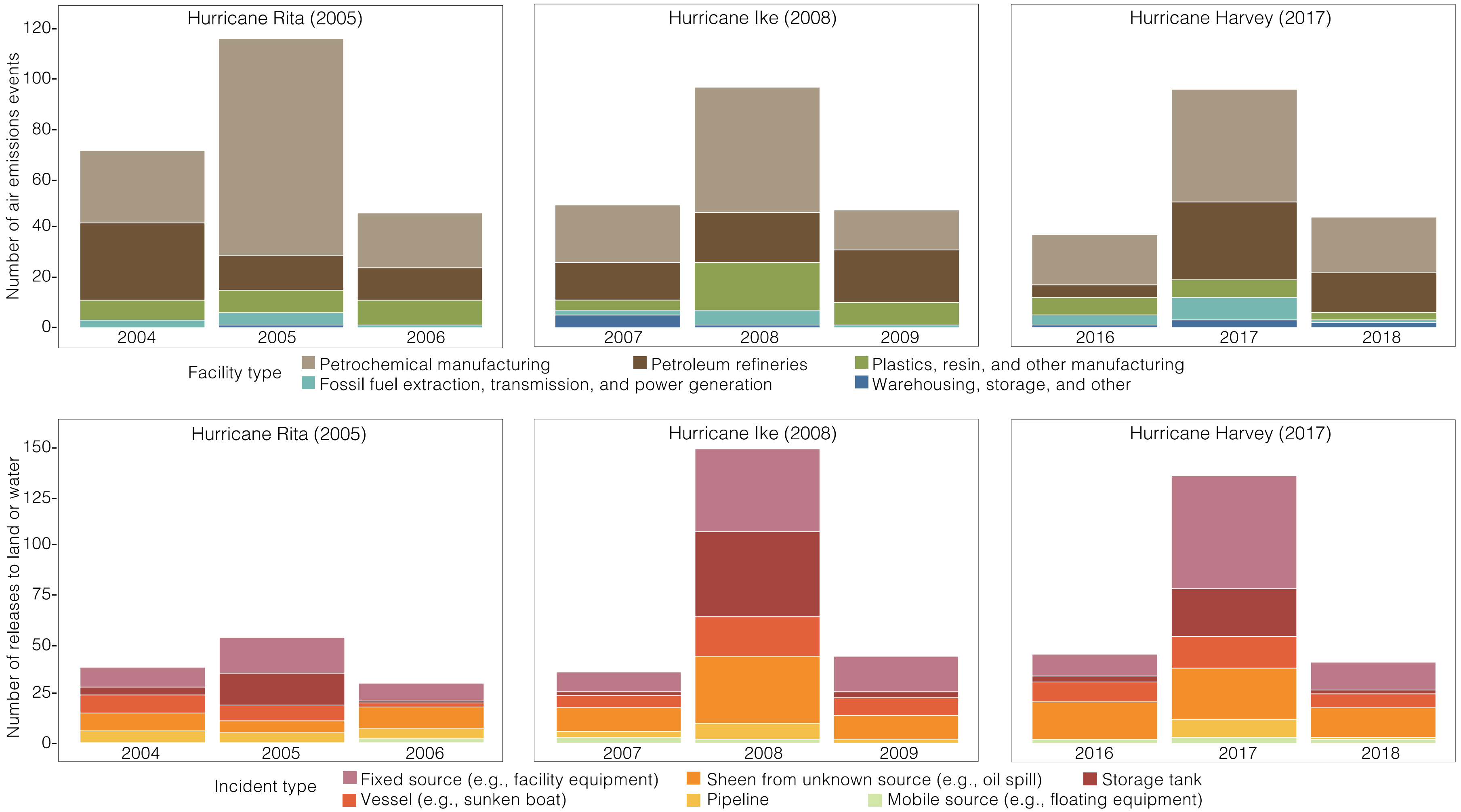

Note. Random days in 2004 and 2006; 2007 and 2009; and 2016 and 2018 include the same number of days as the Hurricane Rita (September 22–October 2, 2005), Ike (September 11–September 21, 2008), and Harvey (August 23–September 5, 2017) periods, respectively. Contaminant releases to land or water for all years were restricted to those with the following causes: flood, hurricane, natural phenomenon, equipment failure, sinking vessel, or unknown causes.

Figure S2. Pounds of excess air contaminants released during Hurricanes Rita, Ike, and Harvey compared to reference periods and random samples

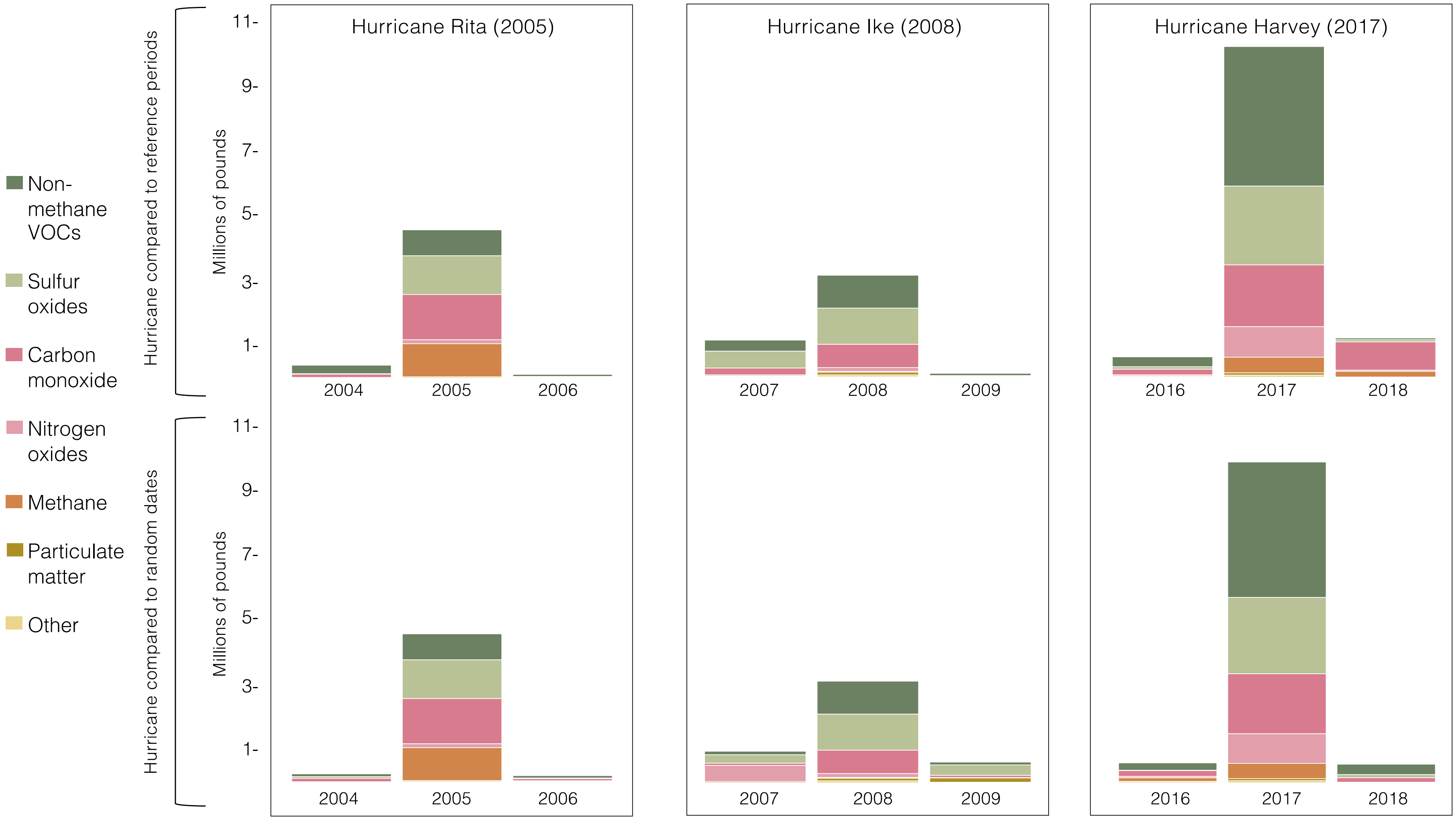

Note. VOC= volatile organic compounds. Reports of carbon dioxide releases were omitted for legibility due to one release of more than 53 million pounds in 2018. Contaminants grouped as “other” include hydrogen, hydrogen sulfide, ammonia, acetone, and lead, among others.
